# Transgenic Rabbits Expressing Ovine PrP Are Susceptible to Scrapie

**DOI:** 10.1371/journal.ppat.1005077

**Published:** 2015-08-06

**Authors:** Pierre Sarradin, Céline Viglietta, Claude Limouzin, Olivier Andréoletti, Nathalie Daniel-Carlier, Céline Barc, Mathieu Leroux-Coyau, Patricia Berthon, Jérôme Chapuis, Christelle Rossignol, Jean-Luc Gatti, Maya Belghazi, Valérie Labas, Jean-Luc Vilotte, Vincent Béringue, Frédéric Lantier, Hubert Laude, Louis-Marie Houdebine

**Affiliations:** 1 INRA-Université de Tours, UMR1282, Infectiologie et Santé Publique, ISP, Nouzilly, France; 2 INRA, UE1277, Plate-Forme d’Infectiologie Expérimentale, PFIE, Nouzilly, France; 3 INRA-CNRS-ENVA, UMR1198, Biologie du Développement et Reproduction, BDR, Jouy-en-Josas, France; 4 UMR INRA ENVT 1225, Interactions Hôtes Agents Pathogènes, Toulouse, France; 5 INRA, UR892, Virologie Immunologie Moléculaires, Jouy-en-Josas, France; 6 INRA- CNRS-UNS, UMR1355, Institut Sophia Agrobiotech, ISA, Sophia Antipolis, France; 7 INRA, UMR INRA85, UMR CNRS 7247, Université de Tours, Institut Français du Cheval et de l’Equitation, Physiologie de la Reproduction et des Comportements, Plate-forme d’Analyse Intégrative des Biomolécules, Nouzilly, France; 8 CNRS-Aix-Marseille Université, UMR7286, Centre de Recherche en Neurobiologie et Neurophysiologie de Marseille, CRN2M, Marseille, France; 9 INRA, UMR1313, Génétique Animale et Biologie Intégrative, Jouy-en-Josas, France; Dartmouth Medical School, UNITED STATES

## Abstract

Transmissible spongiform encephalopathies (TSEs) are a group of neurodegenerative diseases affecting a wide range of mammalian species. They are caused by prions, a proteinaceous pathogen essentially composed of PrP^Sc^, an abnormal isoform of the host encoded cellular prion protein PrPC. Constrained steric interactions between PrP^Sc^ and PrP^C^ are thought to provide prions with species specificity, and to control cross-species transmission into other host populations, including humans. Transgenetic expression of foreign PrP genes has been successfully and widely used to overcome the recognized resistance of mouse to foreign TSE sources. Rabbit is one of the species that exhibit a pronounced resistance to TSEs. Most attempts to infect experimentally rabbit have failed, except after inoculation with cell-free generated rabbit prions. To gain insights on the molecular determinants of the relative resistance of rabbits to prions, we generated transgenic rabbits expressing the susceptible V^136^R^154^Q^171^ allele of the ovine *PRNP* gene on a rabbit wild type *PRNP* New Zealand background and assessed their experimental susceptibility to scrapie prions. All transgenic animals developed a typical TSE 6–8 months after intracerebral inoculation, whereas wild type rabbits remained healthy more than 700 days after inoculation. Despite the endogenous presence of rabbit PrP^C^, only ovine PrP^Sc^ was detectable in the brains of diseased animals. Collectively these data indicate that the low susceptibility of rabbits to prion infection is not enciphered within their non-PrP genetic background.

## Author Summary

Prions are infectious pathogens causing irremediably fatal neurodegenerative diseases in human and in farmed or wild animals. They are formed from abnormally folded assemblies (PrP^Sc^) of the host-encoded prion protein (PrP^C^). Different PrP^Sc^ conformational variants or ‘strains’ can propagate in the same host, giving distinct biological phenotypes. Like other pathogens, prions can transmit from one species to another, representing a zoonotic risk. A barrier, commonly referred to as “species barrier”, limits prion cross-species transmission. This barrier is supposed to reflect the steric incompatibility between invading prion PrP^Sc^ and PrP^C^ of the infected host. Rabbit is one of the species that exhibit a pronounced resistance to prions. To gain insights on the molecular determinants of the relative resistance of this species to prions, we generated transgenic rabbits expressing sheep PrP^C^ and assessed their experimental susceptibility to sheep scrapie prions, as routinely done with transgenic mouse models of prion disease. All transgenic rabbits developed a typical prion disease within 200 days, whereas wild type rabbits remained healthy more than 700 days after inoculation. These data indicate that the low susceptibility of rabbits to prion infection is not enciphered within their non-PrP genetic background.

## Introduction

Scrapie in small ruminants, bovine spongiform encephalopathy (BSE) in cattle, chronic wasting disease (CWD) in cervids, transmissible mink encephalopathy (TME) or Creutzfeldt-Jakob disease (CJD) in humans belong to a group of fatal neurodegenerative diseases referred to as transmissible spongiform encephalopathies (TSEs) or prion diseases [1]. TSEs are characterized by the accumulation in the central nervous system (CNS) of a ß-sheet enriched, protease-resistant and aggregated isoform (PrP^Sc^) of the host encoded cellular prion protein (PrP^C^). During TSE pathogenesis, PrP^Sc^ seeds, acquired through infection or arising from spontaneous conversion of wild type or mutant PrP^C^, are believed to template the conformational change of host PrP^C^ to nascent PrP^Sc^ forms. This autocatalytic polymerization process leads to deposition of injurious deposits into the brain. PrP^Sc^ particles are thought to be the major if not the sole component of TSEs infectious agent or prion [2]. Distinct strains of prions are recognized phenotypically in a given host species. They cause TSEs with specific phenotypic traits, including time course to disease, neuropathological features and PrP^Sc^ biochemical properties. There is compelling evidence that prion strain diversity reflects stable differences in PrP^Sc^ conformations, at the level of the tertiary and/or quaternary structure [3–5].

A wide range of mammals like ruminants, pigs, rodents, carnivores or primates can be naturally and/or experimentally infected with prions. Prions are usually easy to transmit between individuals of the same species. Prions can also transmit between species, as exemplified by the emergence of variant CJD, following dietary exposure of humans to BSE prions. However, such events are restricted by a so-called ‘species’ or ‘transmission’ barrier, the strength of which depends essentially on interactions between host PrP^C^ and the infecting prion strain type(s) [3, 4]. The force of the transmission barrier is classically gauged by the appearance of disease-specific, clinical signs and/or PrP^Sc^ in the brain and, sometimes, extraneural tissues of the new host. Their concomitant absence would usually suggest a resistance to infection or a disease incubation time exceeding that of the exposed host life span [6–9]. Rabbits have long been recognized as one of the best examples of a species refractory to TSEs agents as attempts to transmit Kuru, CJD, sheep scrapie, TME isolates and mouse adapted scrapie strains repeatedly failed [6, 10]. In marked contrast, cell-free PrP^Sc^-templated conversion of PrP^C^ by assays such as the protein misfolding cyclic amplification (PMCA, [11]) revealed that rabbit PrP^C^ was fully convertible into rabbit PrP^Sc^ by seeds from scrapie infected, BSE and mouse scrapie brain sources ([12–14]). Yet, the amplified agents propagated with limited success in rabbits [12], leading support for the hypothesis that rabbit may barely develop clinical TSEs.

Numerous transgenetic studies in mice have demonstrated that mouse resistance to foreign prions can be abrogated by introducing in the murine genome the corresponding *PRNP* gene (the gene encoding PrP^C^). Transgenes can be introduced in animals knocked out for their own *Prnp* gene or on a murine wild-type background (reviews: [15, 16]). Vidal et al. demonstrates that transgenic mice expressing rabbit PrP are permissive to a broad panel of TSE sources from different species [14], strongly advocating for the full convertibility of rabbit PrP^C^ into disease-associated isoforms. While transgenesis on a murine *Prnp* knockout background usually abolishes the transmission barrier, co-expression of the transgene with the wild-type *Prnp* gene can interfere with prion replication [17–19]. In sheep, different *PRNP* alleles tightly modulate the incidence and pathogenesis of classical scrapie. Three codons (for amino acids at positions 136, 154, and 171) act as major determinants, the V_136_R_154_Q_171_ allele conferring the highest susceptibility [20–22]. Experimental transmission of scrapie prions was markedly improved in transgenic mice overexpressing this allele [3, 23].

In this study, we report that rabbits engineered to express transgenetically the V_136_R_154_Q_171_ ovine *PRNP* allele develop a TSE syndrome upon experimental inoculation with scrapie. Disease occurred at full attack rate, with no apparent interfering effect of the rabbit wild-type PrP^C^.

## Material and Methods

### Ethics statement

All the experiments involving animals were done in strict accordance with the European Community Council Directive 86/609/EEC. The French committee for GMO,-formerly the governmental "Commission de Génie Génétique" that belonged to the French Ministry for Education, Research and Technology-, has approved the creation and experimental infection with prions of the rabbit transgenic animal model. The agreement number is 2992-II. It was issued the 12^th^ of November 1998.

### Generation of transgenic rabbits

A fragment of the sheep genome containing the V_136_R_154_Q_171_ allele of the *Prnp* gene was cloned in a bacterial artificial chromosome (BAC) vector previously used to generate the tg338 mouse line [23, 24]. This 125 kb insert, including 40 kb upstream and 60 kb downstream of the *PRNP* gene, was purified following a Not1 digestion and microinjected into New Zealand White rabbit embryos. Approximately 700 hundred embryos (from 41 donor females) were microinjected (mostly in the male pronuclei). 600 embryos were implanted into 30 pseudo-pregnant females. There were 28 live births, of which one was transgenic, as identified by PCR. Rabbit DNA was extracted by digesting ear fragments using proteinase K-SDS method. Digestion was followed by a DNA precipitation by ethanol. The two primers used to identify the sheep *PRNP* gene were TAGGCAGTTGGATCCTGGTT and CCCTATCCTACTATGAGAAA. The primers used to identify the control endogenous αS1-casein gene were CACTCCCTTGTTGAAAACTCTCCTCAG and ATTTTGTGGTTTCAGATCAACCAATAGG.

### RT-qPCR analyses

To estimate the concentration of ovine and rabbit *PRNP* RNA in ovine PrP transgenic rabbits (TgOv), RT-qPCR analyses were performed on 3 animals per line, as previously described [25]. Two micrograms of total RNA (extracted from brain tissue) were reverse-transcribed with random adapters, following the manufacturer’s instructions [25]. Quantification was achieved by SYBR Green quantitative PCR (Applied Biosystems) using sets of primers specific to the *PRNP* ovine and rabbit sequences, resulting in the amplification of 100 bp long fragments, respectively. Two sets of primers were used for rabbit *PRNP*, owing the existence of two variants (with or without exon 2). The sequences of the specific primers were as follows: forward primer: 5’- TCATGGTGAAAAGCCACATAGG-3’, reverse primer: 5’- CCTCCGCCAGGTTTTGGT-3’ for ovine *PRNP*; forward primer: 5’-TCCTCTCGGCAGCTGTCAT-3’, reverse primer: 5’-GCTTCGGCCGCTTCTTG-3’ for Rabbit PRNP without exon 2; forward primer: 5’-AGAGGCCCCAGTCCAGTGTA-3’; reverse primer: 5’-CACTCCACGTGGCCACAA-3’ for Rabbit *PRNP* with exon 2. These primers were chosen so that they are located in different exons. The primers for β-actin were as follows: forward: 5’- CCGCATGCAGAAGGAGATCA-3’; reverse: 5’-AGAGCGAGGCCAGGATGGA-3’. For each sample, RNA concentration normalization was achieved using RT-qPCR on β-actin, as previously described [24]. It is thus given by the formula 2^(CtPRNP-Ctβ-actin)^.

### Scrapie inoculation of transgenic rabbits

Eighteen tgOv rabbits (9 males and 9 females) and 6 wild type (WT) rabbits (3 males and 3 females) aged 103 to 132 days (all bred at INRA Nouzilly) were selected for the experiment. Among them, twelve individually identified tgOv animals and six WT rabbits were injected with 50 μL of a 1% (wt/vol in 5% glucose) brain homogenate from tg338 mice infected with the LA21K *fast* strain. As controls, six tgOv animals were injected similarly with 50 μL of a 10% brain homogenate from a healthy tg338 mouse.

The inocula were prepared in a class II microbiological cabinet using disposable equipment, with strict safety rules, and immediately inoculated to the animals by the intracerebral route, at the level of the right parietal cortex (depth: 1 cm). Inoculations were performed under general anesthesia by injecting a mixture of xylazine (Rompun, Bayer, France) and ketamine (Imalgene, Merial, France) by the intramuscular route. All inoculations were carried out in compliance with ethics and animal welfare according to regulation requirements. The LA21K *fast* strain has been obtained through serial transmission and biological cloning by limiting dilutions of the Langlade field scrapie isolate (INRA Toulouse [26]) to tg338 mice. The LA21K *fast* infectious titer is 109.4 50% lethal doses (LD50)/g of tg338 brain [26].

Rabbits were housed in individual cages, in a dedicated biosafety level-3 facility. They were monitored using a video system and monitored daily for clinical signs and food consumption, by different investigators. Any death arising during the experiment was recorded and animals were necropsied. At clinical stage, rabbits were sacrificed by carbon dioxide suffocation and autopsied for brain and spleen collection. One half of each brain and spleen was stored at –20°C for immunoblotting and biochemical analyses, while the other half of organs was fixed in neutral-buffered 4% formalin for 1 week before paraffin-embedding for immunohistochemistry and histology.

### Immunoblotting

**Fig 1:**
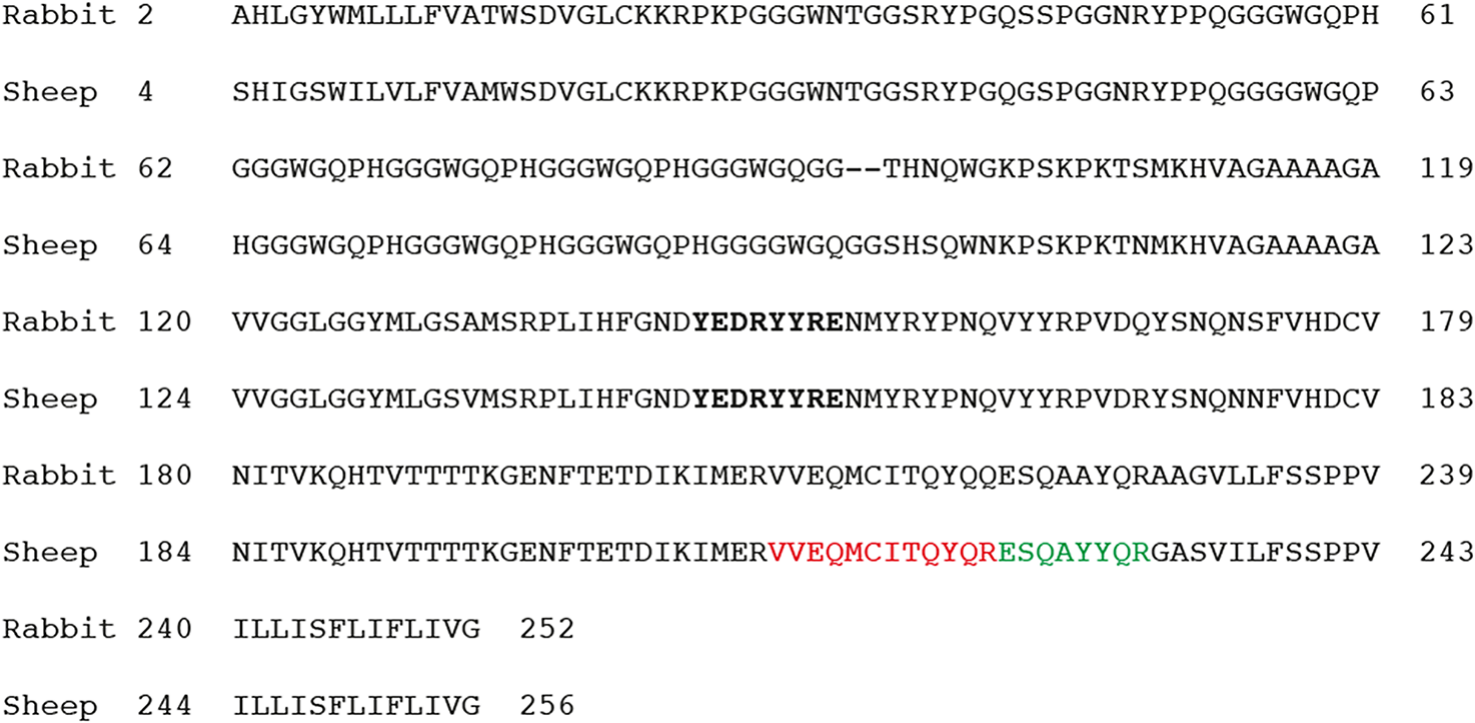
PrP amino acid sequences from New Zealand White rabbit and the sheep (VRQ allele). Fragments identified by mass spectrometry in the brains of scrapie-diseased tgOv rabbit and specific of the sheep sequence are colored. Bold: Sha31 anti-PrP epitope.

Brains and spleens were analyzed for the presence of either PrP^C^ or PrP^Sc^ by Western blotting as previously described [27]. Briefly, homogenates were prepared with a tissue homogenizer (Precellys, Bertin Technologies, France) in a 5% glucose solution. PrP was purified and concentrated using the Bio-Rad TeSeE purification kit. When needed, suspensions were treated with 100 μg/mL of proteinase K (Roche diagnostics, Germany) for 30 min at 37°C and the final pellet was suspended in Laemmli buffer. Samples were denatured at 100°C for 10 minutes, centrifuged at 20 000 g for 5 minutes and the supernatants were run on 12% SDS-PAGE gels. When two-dimensional gel electrophoresis was performed, as previously described [28], the second dimension was run on a 6–16% linear gradient SDS-PAGE. After transfer onto nitrocellulose membranes, samples were probed with Sha31 anti-PrP monoclonal antibody (generous gift of J. Grassi, CEA, Saclay, France, [29]), which binds to the YEDRYYRE amino acid sequence of the PrP protein (amino acid (aa) residues 146 to 153 of the rabbit PrP, Fig 1). This step was followed by the addition of a horseradish peroxidase-conjugate. Peroxidase activity was revealed using a chemiluminescent substrate (SuperSignal West Dura, Pierce, USA), and the signals were captured with a digital imager (Fluorchem 8900, Alpha Innotech, USA) or GeneGnome digital imager (Syngene, Frederick, Maryland, United States). The PrP levels and glycoforms ratios were quantified with the GeneTools software.

Expression levels of PrP^C^ in brains and spleens of rabbits and sheep were compared. Immunoblots revealed with the mAb Sha31 were quantified using the Alpha-Ease software (Alpha-Innotech, USA). Brain samples were diluted and PrP amounts were calculated according to a dilution curve of full-length recombinant ovine PrP (a generous gift of D. Marc, INRA, Tours, France), used as a reference.

### Immunohistochemistry

Rabbit brain sections (5 μm thick) were treated as described previously [30]. Briefly, after being deparaffinised and rehydrated, tissue sections were incubated in 98% formic acid (MERCK) for 30 min at room temperature, then autoclaved for 15 min at 121°C in 10 mM citrate buffer (pH 6.1) and allowed to cool for 20 min. Sections were then subjected to a 15 min proteolysis at 37°C with 20 μg/mL of proteinase K. Endogenous peroxidase was inhibited using 0.3% hydrogen peroxide in methyl alcohol for 30 min at room temperature. Immunostaining was performed on a DAKO Autostainer according to the manufacturer’s instructions using the mouse monoclonal anti-PrP antibody 2G11 (gift from J. Grosclaude, INRA, Jouy-en-Josas, France, [30]) as primary antibody, followed by the DAKO EnVision+ System labelled polymer-HRP anti-mouse with 3, 3’-diaminobenzidine (DAB) as chromogen. After immunostaining, sections were counterstained with Mayer’s haematoxylin and cover-slipped. The 2G11 antibody was selected for optimal results with TSE-infected-tgOv rabbit brains without any background staining on tissue sections from both uninfected-tgOv and–WT rabbits.

### Paraffin-embedded tissue blot (PET blot)

PET blot were performed using a method previously described [31]. Immunodetection was performed with Sha31 monoclonal antibody (4 μg/mL), followed by application of an alkaline phosphatase labeled secondary antibody (Dako, 1/500 final dilution). Enzymatic activity was revealed using NBT/BCIP substrate chromogen.

### Histology

Paraffin-embedded sections were mounted on glass microscope slides and stained with hematoxylin and eosin.

### Mass spectrometry

The equivalent of 6 mL of 20% brain homogenate from tgOv and WT rabbit, treated or not with proteinase K and denatured (see above) were resuspended in 100 mM NaCl, 10 mM EDTA, 10 mM TrisHCl pH 7.8, 0.5% DOC, 0.5% Igepal (Sigma). Monoclonal antibody Sha31 coupled to magnetic beads (Dynabeads M-280 Tosylactivated, Dynal) was added at the rate of 100 μL beads/240 mg tissue equivalent and reacted for 2 hours at 37°C. Beads were collected with a magnet, washed twice with PBS buffer and denatured in Laemmli buffer (10 min at 99°C). Samples were loaded on a 12% acrylamide gel, before electrophoresis and either immunoblotted (60 mg of tissue equivalent) or silver stained (1140 mg of tissue equivalent). The silver stained gels were compared to the western blot and protein bands that were at the same molecular masses than the PrP^Sc^ reactive bands were cut, rinsed and then reduced with dithiothreitol and alkylated with iodoacetamide. Samples were incubated overnight at 37°C with 12.5ng/μl trypsin (sequencing grade, Roche, Meylan, France) in 25 mM NH4HCO3 [28]. Tryptic peptides were analyzed by nanoLC-MS/MS with Q-q-TOF and Linear Ion trap.

For CapLC system coupled to Q-TOF Ultima Global (Waters Micromass, Manchester), the digested peptides were loaded on a precolumn (300μm i.d x 5mm, packed with C18 PepMap, LC Packings, Dionex) and desalted. Peptide separations were conducted on a C18 column (Atlantis dC18, 75mm I.D x 150 mm Nano Ease, Waters). Peptides were eluted with a 5–60% linear gradient with water/acetonitrile 98/2 (v/v) containing 0.1% formic acid in buffer A and water/acetonitrile 20/80 (v/v) containing 0.1% formic acid inbuffer B. Mass data were acquired using one MS survey followed by MS/MS scans on the 3 most intense ions detected. Data were processed using ProteinLynx Global server 2.2. The peptide and fragment masses were matched in database (nrNCBI) using MASCOT software (http://www.matrixscience.com). The mass tolerance was 0.2 Da for both precursor and fragment ions.

For Ettan MDLC system (GE Healthcare, Germany) coupled to LTQ Linear Ion Trap Mass Spectrometer (Thermo Electron, US), each sample was desalted using Zorbax 300-SB C_18_ trap column, 300μm i.d x 5 mm (Agilent Technologies, Germany). Peptide separations were conducted on a Zorbax 300-SB C_18_ column, 75 μm i.d x 150 mm (Agilent Technologies, Germany). Buffer A consisted of water with 0.1% formic acid while buffer B was 84% acetonitrile with 0.1% formic acid. Separation was performed by applying gradient of 15–55% B for 60 minutes at a flow rate of 400 nL/min. Mass data were acquired using one MS survey (m/z 500–2000) followed by MS/MS scans on the 3 most intense ions detected using Collision Induced Dissociation fragmentation mode. Identification was then performed with Bioworks 3.2 (Thermo Finnigan, San Jose, CA) software. MS/MS spectra were searched against the non-redundant Uniprot database (2006_12) and analysed using TurboSEQUEST (Thermo Finnigan, San Jose, CA). Search parameters included differential amino acid mass shifts for oxidized methionine (+16 Da) and carbamidomethylation on cystein (+57 Da). The output data were evaluated in term of Xcorr magnitude up to 1.7, 2.2 and 3.5 for charge states 1+, 2+ and 3+, respectively.

### Cell culture

Confluent Rov cells (P2FJ6 clone, [26, 32]) were grown for 2 days in single wells of 12-well plates. Rov cells were incubated in culture medium containing 10 μL of 20% brain homogenate from tgOv rabbits, WT rabbits and tg338 mice challenged with LA21K *fast* prions, and from uninfected tgOv rabbits. After 2 days, the medium was removed; the cells were rinsed in phosphate-buffered saline (PBS) and split into 25 cm^2^ flasks. Each week, one flask was used for subpassaging, whereas another was used to prepare a cell lysate for PrP content analysis (see above). The total protein content was estimated by using a protein assay kit (bicinchoninic acid assay (BCA); Pierce).

### Accession number

The Swiss-Prot accession numbers for the proteins mentioned in the text are sheep (P23907) and rabbit PrP (Q95211).

## Results

### Production and characterization of transgenic rabbits

One transgenic rabbit founder animal was obtained following microinjection of an ovine BAC DNA insert encompassing the entire *PRNP* transcription unit. This insert has already been used to produce various mouse transgenic lines that express the PrP^VRQ^ allele [23]. For animal production, the tgOv transgenic founder was mated with a WT rabbit. Transmission of the transgene was of about 50% indicating that the rabbit founder was not a mosaic. The transgenic F1 rabbits were mated with WT rabbits giving birth to 39 offspring including 50% of heterozygous transgenic rabbits and 50% of non-transgenic control rabbits. Health of the rabbits did not appear to be affected by the presence of the ovine *PRNP* transgene (period of observation > 700 days).

The concentration of ovine *PRNP* RNA relative to that of rabbit *PRNP* RNA was estimated by RT-qPCR analyses on brain tissue extracts of tgOv rabbits and WT rabbits. In WT and tgOv rabbits, two rabbit *PRNP* RNA variants were found, as expected, resulting from the splicing (or not) of exon 2. The values were cumulated to obtain the total concentration of rabbit *PRNP* transcripts. In tgOv rabbits, ovine *PRNP* RNA levels were 1.5–2 fold higher than those of rabbit *PRNP* RNA (n = 3 rabbits analyzed). Rabbit *PRNP* RNA levels were similar between WT and TgOv rabbits, suggesting that expression of ovine *PRNP* has no interfering effects on the transcription of rabbit *PRNP*.

**Fig 2:**
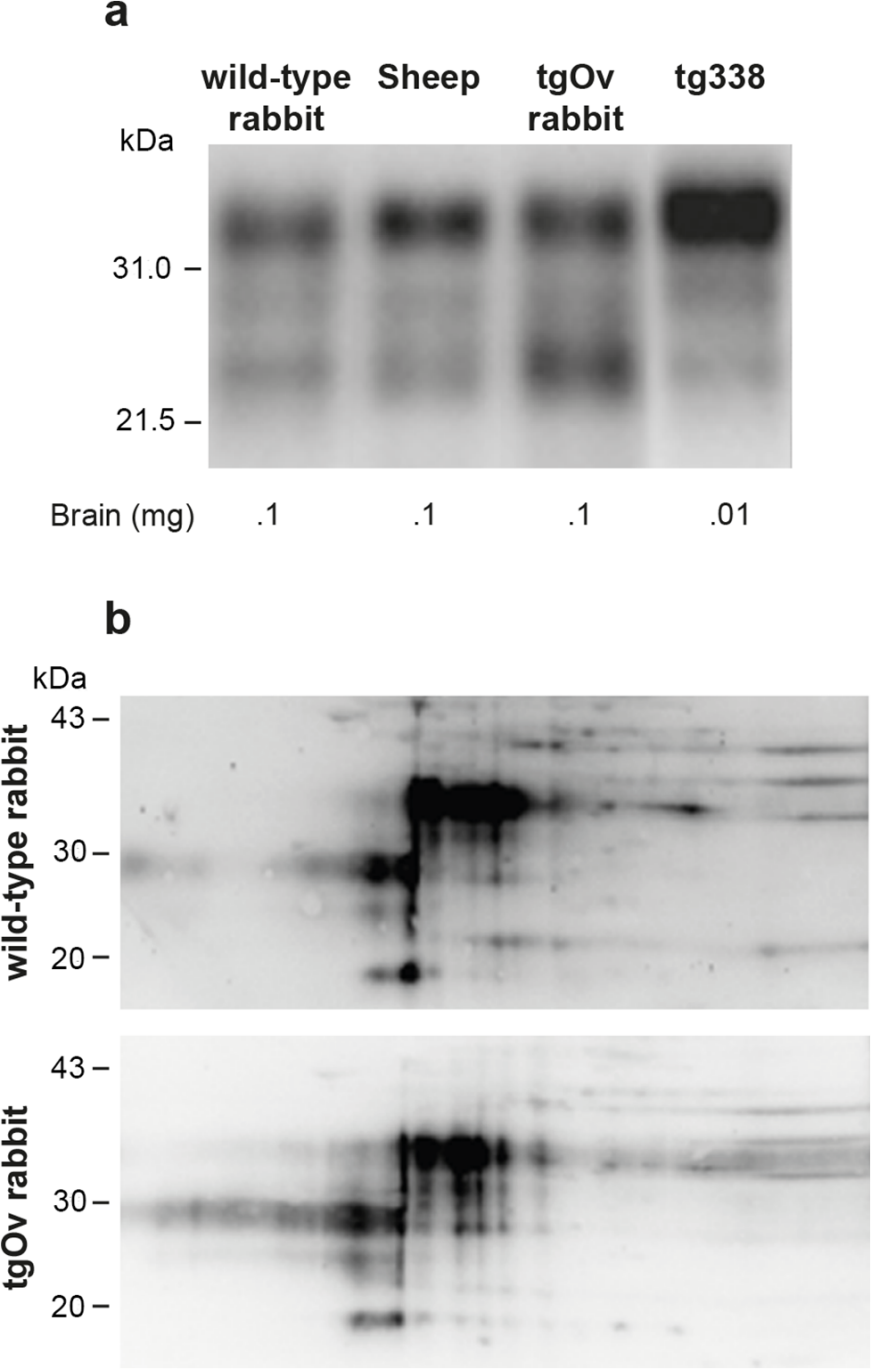
Imunoblot analyses of PrP in the brains of wild-type and ovine PrP transgenic rabbits. (a) PrP^C^ electrophoretic pattern and level of expression in the brain of wild-type, sheep (VRQ allele), tgOv rabbit and tg338 mice. The amounts of material loaded are indicated. (b) Two-dimensional electrophoretic gel analysis of PrP^C^ from wild-type and tgOv rabbit. The equivalent of 1mg of brain extract was used for comparison (Acidic side at left). Blots were probed with Sha31 anti-PrP antibody.

In the absence of rabbit-specific anti-PrP antibody, it was not possible to quantify the relative expression levels of ovine versus endogenous rabbit PrP^C^ in tgOv rabbits. Immunoblots analyses indicate that the total levels of PrP^C^ in brain of tgOv rabbits were approx. 1.5–2 fold higher than those found in the brain of their WT counterparts or sheep carrying the VRQ allele (Fig 2A), in agreement with the transcriptional analysis. TgOv rabbits expressed about 50 fold more PrP^C^ in the brain than in the spleen, which compares with the ratio of about 60 found in WT rabbit.

Two-dimensional gel electrophoresis was performed to determine whether any change in the isoforms pattern of PrP^C^ was visible between WT and tgOv rabbits. Equivalent amounts of brain extracts were separated and transferred to nitrocellulose and probed with the Sha31 antibody (Fig 2B). Both extracts gave similar 2D patterns and none of the isoforms appeared specific or quantitatively different between the two types of rabbits.

### Transgenic rabbits expressing ovine PrP are susceptible to scrapie

**Fig 3:**
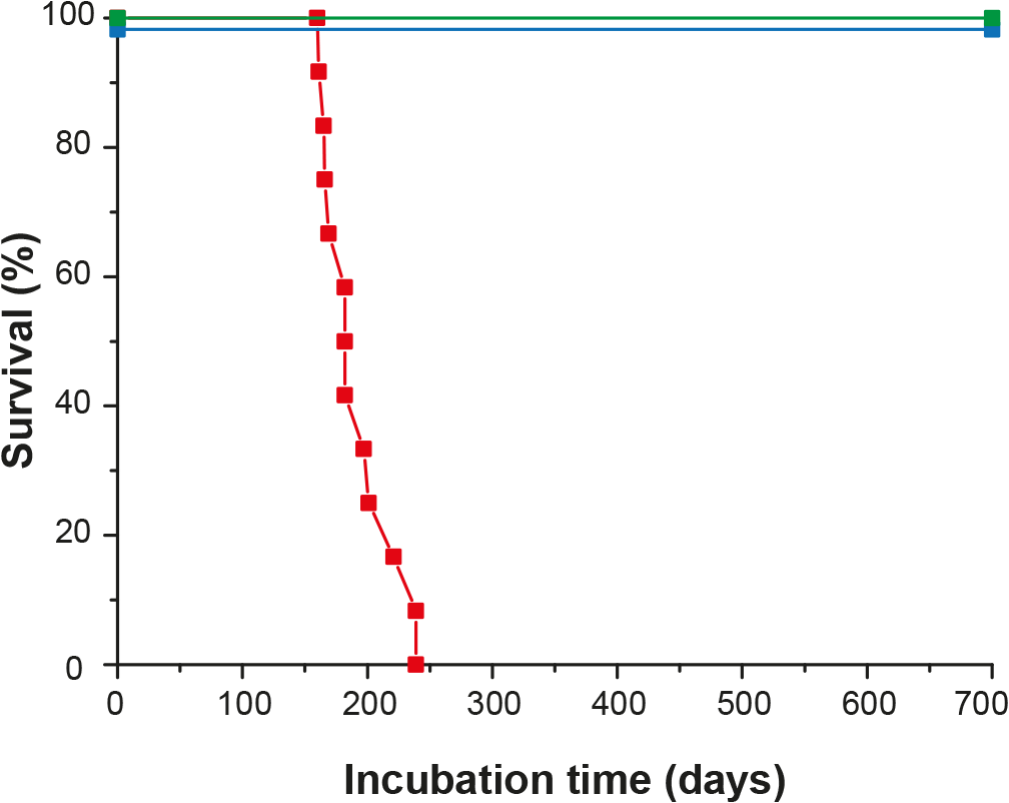
Survival time of ovine PrP transgenic rabbits infected with scrapie. Kaplan-Meier survival plots of wild-type and tgOv rabbits inoculated intracerebrally with LA21K *fast* scrapie prions or with healthy brain homogenates. Animals were monitored for clinical signs and analyzed for the presence of PrP^Sc^ either by WB or immunohistochemistry. LA21K *fast*-inoculated tgOv rabbits: red symbol; LA21K *fast*-inoculated-WT rabbits: blue symbol; mock-inoculated tgOv rabbits: green symbol.

TgOv and WT rabbits were intracerebrally inoculated with LA21K *fast* scrapie strain, a fast strain that kills tg338 mice in less than 2 months [26].This agent induced a neurological disease in all inoculated tgOv rabbits (*n = 12*). The behavioral and clinical signs were invariant from animal to animal. The first behavioral signs were a drop in food consumption and restlessness. Early neurological signs (referred to as moderate in the S1 Video) were characterized by amaurosis and decreased time of random exploration in the cage. With disease progression, more severe neurological and behavioral signs were progressively observed, including loss of balance, disordered gait, paparesis, drop in food consumption and more severe amaurosis, (S2 Video). Animals were euthanized as soon as at least 3 of these signs were recorded. Neither pruritus nor tremor was observed. The clinical phase lasted less than 2 weeks. Animals were euthanized between 161 and 239 days post-inoculation (mean ± SEM incubation time: 192 ± 8 days; Fig 3). None of the mock-infected tgOv rabbits (n = 6) or LA21K *fast*-challenged WT rabbits (n = 6) presented any clinical signs during the time course of the experiment (Fig 3, S3 Video). They were euthanized healthy at 701 days post-inoculation, except two animals from each control group that were sacrificed, for comparison purpose with scrapie-sick animals.

**Fig 4:**
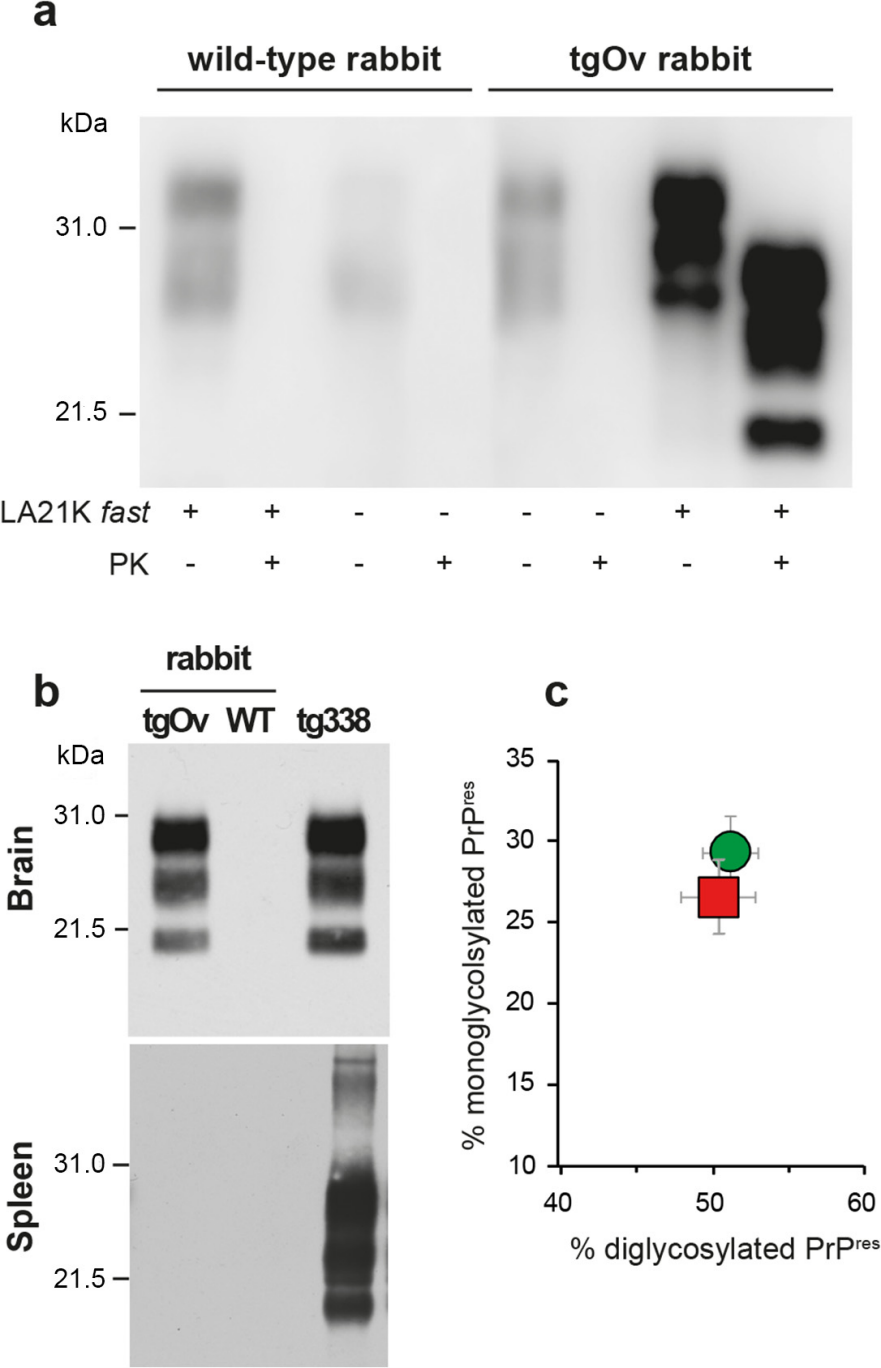
Brain PrP^Sc^ in ovine PrP transgenic rabbit infected with LA21K fast scrapie prions. (a) Western blot analyses of the brain from wild-type and tgOv rabbits mock-infected or inoculated with LA21K *fast* prions for the presence of proteinase K (PK)-resistant PrP^Sc^. The equivalent of 2 mg brain tissue were loaded in lane 1–6, 3 mg in lanes 7 and 8. (b) Electrophoretic pattern and (c) glycoform ratios (plotted as means ± SEM(c)) of PrP^res^ in the brains and spleens of tgOv rabbit and tg338 mice infected with LA21K fast prions. The same amount of brain (0.5 mg) and spleen (3 mg) tissue equivalent was loaded on the gel.

**Fig 5:**
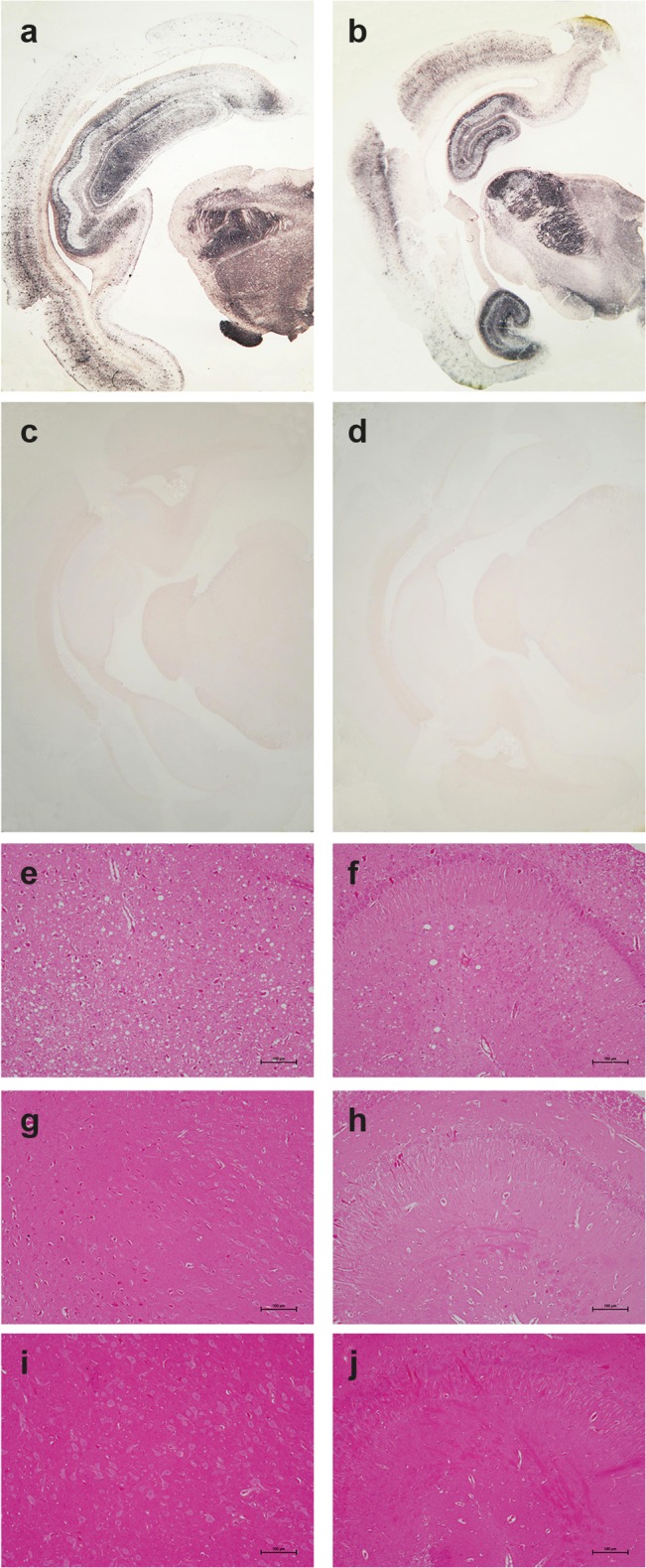
Pathological PrP deposition and vacuolation in the brains of wild-type and tgOv rabbits inoculated with LA21K *fast* scrapie prions. Midbrain sections from tgOv (a-b, e-f) and wild-type (c, g-i) rabbits challenged with LA21K *fast* scrapie prions and from a mock-infected tgOv rabbit (d, i-j). (a-d) PET blot analyses using monoclonal antibody Sha31 showed PrP^res^ accumulation solely in LA21K *fast* challenged tgOv rabbits. (e-j) Hematoxylin and eosin-stained section at the level of the thalamus (e, g, i) and hippocampus (f, h, j) showing vacuolation, predominantly in the thalamus (e) and to a lesser extend in the hippocampus (f) of the LA21K *fast* challenged tgOv rabbits, but none in the control animals (g-j). Scale bar: 100 μm.

The brains of diseased tgOv animals were analyzed by immunoblotting and immunohistochemistry for the presence of PrP^Sc^. Proteinase-K resistant PrP^Sc^ (PrP^res^) was readily detected in all infected tgOv rabbits (Figs 4 and 5), consistent with the efficient transmission. All controls remained PrP^res^ negative (Fig 4A and 4B). In the absence of PK-treatment, PrP^Sc^ was essentially detected as full-length PrP^Sc^ (Fig 4A) as in tg338 brain [33], suggesting absence of endogenous cleavage generating the so-called C2 fragment [33]. Remarkably, LA21K *fast* electrophoretic pattern was conserved in tgOv rabbits with regards to apparent molecular mass and relative proportions of glycoforms (Fig 4B and 4C). None of the animals of the experiment, including positive transgenic rabbits, showed any detectable PrP^res^ deposits in the spleen (Fig 4B). LA21K *fast*, as other *fast* ovine strains is lymphotropic in tg338 mice ([34], Fig 4B). It is likely that disease after intracerebral inoculation has occurred too rapidly in the rabbits to allow centrifugal spreading and replication of LA21K *fast* scrapie prions in the spleen.

PrP^res^ distribution in the brain was examined by PET blot analyses and immunohistochemistry. The greatest levels of PrP^res^ deposits were observed in the thalamus, hippocampus and frontal cortex of scrapie-sick TgOv rabbits (Fig 5A and 5B). Sparse or moderate PrP^Sc^ deposition was seen in the cerebellum, obex and medulla oblongata (Table 1, analysis of 9 animals). Aged, uninfected tgOv rabbits and LA21K *fast*-challenged WT rabbits remained PrP^res^ negative (Fig 5C and 5D).

**Table 1:** Regional distribution of PrP^res^ and vacuolation in the brain of transgenic rabbits expressing ovine PrP challenged with LA21K *fast* prions.

Brain Regions	PrP^res^ deposits	Spongiosis
Cortex	+++	-
Hippocampus	+++	+
Thalamus	+++	++
Cerebellum	+/-	+/-
Obex	+	+/-
Medulla oblongata	+	+

-: no staining, no vacuolation; +/-: low; +: moderate; ++: pronounced; +++, intense staining or spongiosis.

Examination of histopathologic lesions in several brain areas of scrapie-sick TgOv rabbits (5 animals analyzed) indicated that spongiosis was prominent in the thalamus (Fig 5E). Mild spongiosis was also observed in the hippocampus (Fig 5F). Sparse spongiosis was observed in the medulla oblongata, obex and cerebellum (Table 1). The cortex was not vacuolated. There was no evidence of vacuolation in aged, uninfected tgOv rabbits and LA21K *fast*-challenged WT rabbits (Fig 5H–5J).

Collectively, these data indicate that scrapie infected tgOv rabbits exhibited the major clinical, biochemical and neuropathological hallmarks of TSEs.

### Diseased transgenic rabbits accumulate only ovine PrP^Sc^

To determine which of the rabbit or the ovine PrP^C^ had been converted in scrapie-sick tgOv rabbits, immunoprecipitated brain extracts of healthy and infected tgOv rabbits,—treated or not with PK-, were analyzed by two techniques of mass spectrometry. Both analyses allowed detecting PrP fragments in the different gel bands corresponding to the western blot immunoreactive bands. In PK-treated, LA21K *fast* infected tgOv rabbit brains, five fragments were identified: ESQAYYQR; GENFTETDIK; VVEQMCITQYQR; GENFTETDIKIMER; EHTVTTTTKGENFTETDIK. Only one fragment VVEQMCITQYQR was identified in mock-infected tgOv rabbit, while no fragments were obtained from WT rabbits. All these fragments were located within the C-terminal part of the PrP protein and two could be assigned without ambiguities to the sheep PrP sequence (ESQAYYQR; VVEQMCITQYQR; Fig 1). Thus, PrP^res^ molecules that accumulate in the brain of scrapie-sick tgOv rabbits was essentially of ovine origin, suggesting limited conversion, if any of endogenous rabbit PrP^C^ during disease pathogenesis.

### Prions from diseased transgenic rabbits readily infect Rov cells

**Fig 6:**
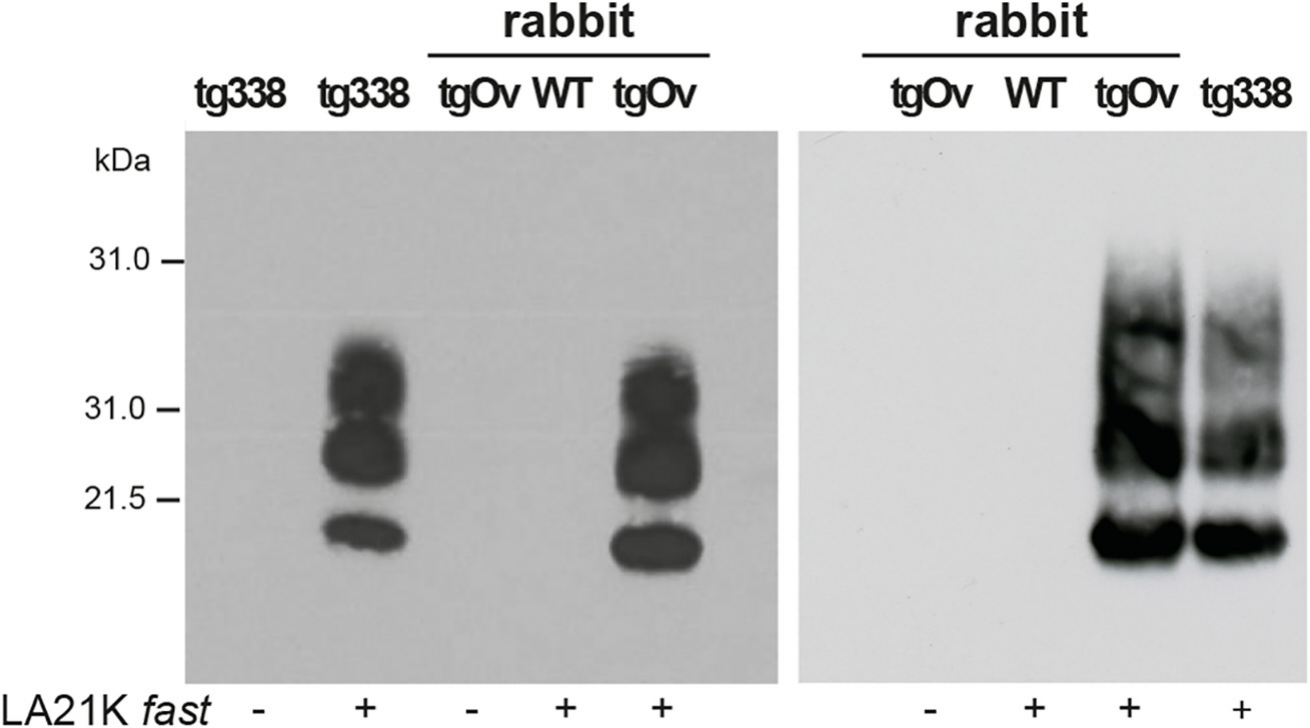
Infection of Rov cells with TgOv- and tg338-passaged LA21K *fast* prions. Western blot analyses for the presence of PrP^res^ in lysates from Rov cells exposed to brain homogenates from WT, tgOv rabbits and tg338 mice that were mock infected or challenged with LA21K *fast* prions. Analysis was performed two (left panel) and four passages (right panel) after initial exposure of the cells. The loaded samples were normalized relative to total protein concentration.

LA21K *fast* scrapie prions can be efficiently passaged in Rov cells expressing ovine PrP [26, 32, 35]. We examined whether the prions produced in the brain of LA21K *fast*-sick tgOv rabbits would infect Rov cells with similar efficacy. Rov cells were exposed to similar amounts of brain homogenate from LA21K *fast*-infected tgOv rabbit and tg338 mice, and grown for up to 4 passages. At each passage, PrP^res^ accumulation was monitored to assess the success of the infection and compare the levels of protein produced. In parallel, cells were exposed to brain extracts from aged, uninfected tgOv rabbits and from LA21K *fast*-inoculated WT rabbits. While immunoblots analyses of PK-digested cell lysates failed to detect PrP^res^ in these controls, cells exposed to LA21K *fast* prions from either tgOv rabbit or tg338 mouse origin accumulated similar levels of PrP^res^ at each passage (Fig 6). LA21K *fast* prions derived from tgOv rabbits and tg338 mice exhibit therefore similar efficacy to infect Rov cells. These data would further sustain the view that the prions produced in the brains of tgOv rabbits are of ovine origin.

## Discussion

The limited number of prion-permissive cell models, the prolonged incubation time in farm species and the low susceptibility of conventional mouse lines to TSEs agents have favored development of mouse transgenesis in the TSE field. Some of the *Prnp* ^0/0^ mouse lines used to demonstrate the key role of PrP^C^ in susceptibility to prions [36] were eventually engineered to (over)express *PRNP* genes from a wide range of mammalian species. These models considerably improved our knowledge on prion diversity, and most particularly on the molecular determinants of the transmission barrier during interspecies prion transmission [3]. A side effect of these studies was that transmission barriers that were considered as strong or ‘absolute’ were essentially abrogated [19, 23, 37, 38]. Here, we applied the same strategy to the rabbit species to clarify the respective roles of rabbit PrP^C^ and non-PrP host factors in their pronounced, albeit not absolute [12], resistance to TSEs. We show that transgenic rabbits expressing a scrapie-susceptible ovine *PRNP* allele develop, at full attack rate, classical hallmarks of TSEs upon inoculation with scrapie prions, including fatal neurological diseases, clinical signs, PrP^res^ deposition and vacuolation in the brain. This demonstrates that rabbits do not bear non-PrP factors that make them intrinsically resistant to prions.

The strategy used to generate the *tg338* mouse line, a model highly susceptible to sheep scrapie sources [3, 23, 26, 34], was transposed to rabbit. At variance with the tg338 mouse line, transgenesis was performed on a wild type rabbit background, as *Prnp* ^0/0^ rabbits are not available, leading to the likely expression of both rabbit and sheep PrP proteins. Analyses of the *PRNP* transcripts indicated that ovine *PRNP* transcripts were present in tgOv rabbit brain at levels 1.5–2 fold higher than those of rabbit *PRNP*. Consistently, expression level of total PrP^C^ in tgOv rabbit brains was 1.5–2 fold higher than that in WT rabbit and sheep. Co-expression of two different prion proteins can have a strong inhibitory effect on the conversion into prions of the transgenic PrP^C^ protein, resulting in either no transmission, or a marked increase of the incubation time or no clinical disease [17–19, 39]. Here, scrapie developed at full attack rate in tgOv rabbits. The molecular, LA21K *fast* strain-specific [3] signature was conserved upon passage to another species expressing the same transgene in a different genetic background. The potential of LA21K *fast* prions to infect Rov cells [26, 32] was unaltered by the intermediate passage onto TgOv rabbits. Collectively, these data support the view that transgenic expression of the ovine PrP^VRQ^ allele abrogated the rabbit species barrier to LA21K *fast* scrapie prions. Demonstrating formally that the species barrier has been fully abrogated and/or that co-expression of rabbit and sheep PrP^C^ had no major interfering effects on scrapie pathogenesis would necessitate further subpassaging on tgOv rabbits to measure a potential reduction in incubation time, if any [3]. This experiment has not been done. However, it can be noticed that the mean incubation time observed at primary passage in tgOv rabbits (<200 days) is within the range of incubation time observed in sheep and transgenic mice expressing physiological levels of ovine PrP upon intracerebral infection, at the same dose, of *fast* scrapie prions ([23, 30]; 140 ± 5 days (6/6) in tg335 mice [23]).

While scrapie-infected tgOv rabbits showed characteristic signs of prion diseases, their WT counterparts remained healthy for more than 700 days, with neither detectable signs nor lesions or PrP^Sc^ deposits. This confirmed with another natural TSEs source the pronounced resistance of the rabbit species to foreign prions [6, 10]. We also showed by mass spectrometry that most if not all of the converted PrP^C^ molecules in the brains of scrapie-sick tgOv rabbits were of ovine origin, indicating that conversion of rabbit PrP^C^ was not favored by the conversion of ovine PrP^C^ at vicinity. Collectively, these data suggest that rabbit PrP^C^ is poorly convertible into LA21K *fast* PrP^Sc^ in vivo. Consistently, cell-free conversion of rabbit PrP^C^ by SSBP/1 prions,-which exhibit a LA21K *fast* phenotype in tg338 mice (mean survival time of 63 ± 1 days in 6/6 tg338 mice on primary passage)-, necessitated a relatively high number of PMCA rounds as compared to other prion sources [12]. To further address the issue of the presence of rabbit PrP^Sc^ in the brain of diseased tgOv rabbits, secondary passage to transgenic mice expressing rabbit PrP^C^ are planned ([12] and the accompanying paper by Vidal et al.).

The common shared view that rabbits were resistant to prion infection was not only attributed to rabbit PrP^C^ sequence but also to its genetic background [12, 40–42]. Vidal and Castilla’s groups demonstrate by using transgenic modeling that rabbit PrP^C^, as many other mammalian PrP^C^, is fully convertible into disease-specific isoforms after infection with a broad panel of TSE sources [14]. Thus, taken separately, rabbit genetic background and rabbit PrP^C^ cannot explain the apparently low susceptibility of rabbits to prion infection. What makes the rabbit species comparatively resistant to prion disease remains to be clarified. On the one hand, the diversity of prion sources inoculated to this species has remained too limited [6, 10] to definitely conclude that rabbits are poor acceptor for prions. The difficulty in identifying TSEs agents able to replicate on certain PrP sequence has been recently exemplified by studies on scrapie prions zoonotic potential. Such evidence (contrarily to a common- shared view) was provided because a panel of diverse scrapie sources was inoculated to human PrP transgenic mice [43]. On the other hand, it is possible that prion disease in rabbit would develop too slowly to be observed, because of a low conversion rate of rabbit PrP^C^. Transgenic modeling with mice expressing PrP at variable levels may help to verify this hypothesis [23, 44].

To summarize, we found that rabbit expressing ovine PrP at near physiological levels can develop a *bona fide* TSE upon infection with scrapie prions. The low susceptibility of rabbits to prion infection is not enciphered within the rabbit genetic background. Owing to its sensitivity and intermediate size, this model may be a valuable tool for studying TSE pathogenesis, most notably prionemia.

## Supporting Information

S1 Video Moderate clinical signs in tgOv rabbits infected with LA21K fast scrapie prions. (MOV)

S2 Video Severe clinical signs in tgOv rabbits infected with LA21K fast scrapie prions. The animals were euthanized at that stage.
(MOV)

S3 Video Absence of neurological signs in age-matched mock-infected tgOv rabbits. (MOV)
